# Autophagy impairment in patients with obstructive sleep apnea modulates intermittent hypoxia-induced oxidative stress and cell apoptosis via hypermethylation of the *ATG5* gene promoter region

**DOI:** 10.1186/s40001-023-01051-4

**Published:** 2023-02-17

**Authors:** Yung-Che Chen, I-Chun Lin, Mao-Chang Su, Po-Yuan Hsu, Chang-Chun Hsiao, Te-Yao Hsu, Chia-Wei Liou, Yu-Mu Chen, Chien-Hung Chin, Ting-Ya Wang, Jen-Chieh Chang, Yong-Yong Lin, Chiu-Ping Lee, Meng-Chih Lin

**Affiliations:** 1 Division of Pulmonary and Critical Care Medicine, Department of Medicine, Kaohsiung Chang Gung Memorial Hospital and Chang Gung University College of Medicine, No. 123, Ta-Pei Rd, Niao-Sung District, Kaohsiung, 83301 Taiwan; 2 Department of Medicine, College of Medicine, Chang Gung University, Taouyan, 33302 Taiwan; 3 Sleep Center, Kaohsiung Chang Gung Memorial Hospital and Chang Gung University College of Medicine, No. 123, Ta-Pei Rd, Niao-Sung District, Kaohsiung, 83301 Taiwan; 4 Department of Pediatrics, Kaohsiung Chang Gung Memorial Hospital and Chang Gung University College of Medicine, Kaohsiung, 83301 Taiwan; 5 Chang Gung University of Science and Technology, Chiayi, 61363 Taiwan; 6 Graduate Institute of Clinical Medical Sciences, College of Medicine, Chang Gung University, Taouyan, 33302 Taiwan; 7 Department of Obstetrics, Kaohsiung Chang Gung Memorial Hospital and Chang Gung University College of Medicine, Kaohsiung, 83301 Taiwan; 8 Department of Neurology, Kaohsiung Chang Gung Memorial Hospital and Chang Gung University College of Medicine, Kaohsiung, 83301 Taiwan; 9 Genomics and Proteomics Core Lab, Department of Medical Research, Kaohsiung Chang Gung Memorial Hospital, Kaohsiung, 83301 Taiwan

**Keywords:** Obstructive sleep apnea, Autophagy, DNA methylation, Mesenchymal stem cell, Intermittent hypoxia with re-oxygenation, Rapamycin

## Abstract

**Background:**

Autophagy is a catabolic process that recycles damaged organelles and acts as a pro-survival mechanism, but little is known about autophagy dysfunction and epigenetic regulation in patients with obstructive sleep apnea (OSA).

**Methods:**

Protein/gene expressions and DNA methylation levels of the autophagy-related genes (ATG) were examined in blood leukocytes from 64 patients with treatment-naïve OSA and 24 subjects with primary snoring (PS).

**Results:**

LC3B protein expression of blood monocytes, and ATG5 protein expression of blood neutrophils were decreased in OSA patients versus PS subjects, while p62 protein expression of cytotoxic T cell was increased, particularly in those with nocturia. *ATG5*, *ULK1*, and *BECN1* gene expressions of peripheral blood mononuclear cells were decreased in OSA patients versus PS subjects. *LC3B* gene promoter regions were hypermethylated in OSA patients, particularly in those with excessive daytime sleepiness, while *ATG5* gene promoter regions were hypermethylated in those with morning headache or memory impairment. LC3B protein expression of blood monocytes and DNA methylation levels of the *LC3B* gene promoter region were negatively and positively correlated with apnea hyponea index, respectively. In vitro intermittent hypoxia with re-oxygenation exposure to human THP-1/HUVEC cell lines resulted in LC3B/ATG5/ULK1/BECN1 down-regulations and p62 up-regulation along with increased apoptosis and oxidative stress, while rapamycin and umbilical cord-mesenchymal stem cell treatment reversed these abnormalities through de-methylation of the *ATG5* gene promoter.

**Conclusions:**

Impaired autophagy activity in OSA patients was regulated by aberrant DNA methylation, correlated with clinical phenotypes, and contributed to increased cell apoptosis and oxidative stress. Autophagy enhancers may be novel therapeutics for OSA-related neurocognitive dysfunction.

**Supplementary Information:**

The online version contains supplementary material available at 10.1186/s40001-023-01051-4.

## Introduction

Obstructive sleep apnea (OSA) is characterized by recurrent upper airway collapse during sleep leading to sleep fragmentation and chronic intermittent hypoxia with re-oxygenation (IHR) in association with overt oxidative stress and systemic low-grade inflammation. OSA is an important risk factor for a variety of co-morbidities, including hypertension, strokes, atrial fibrillation, ischemic heart disease, heart failure, diabetes mellitus, chronic kidney disease, and neurocognitive dysfunction, as well as higher mortality [1, 2]. The mainstream treatment for OSA is continuous positive airway pressure (CPAP) devices, whereas meta-analyses of the recent randomized control trials have failed to demonstrate convincing evidence for its benefits for major cardiovascular outcomes, except for OSA patients with hypertension or stroke [3]. Since the required level of CPAP adherence for the effectiveness may be too high for most patients to achieve, it is important to identify the best pharmacological approach to counteract the key intermediary mechanisms responsible for OSA-related adverse consequences.

Autophagy is an evolutionarily conserved catabolic process that maintains cellular homeostasis by degrading and recycling damaged organelles and toxic agents through an autophagosomal–lysosomal pathway [4]. Autophagy is up-regulated in response to oxidative stress, helping to restore intracellular homeostasis by disposing a number of harmful molecules, such as mis-folded proteins overflowing from endoplasmic reticulum stress, cytosolic proteins damaged by reactive oxygen species (ROS), or dysfunctional mitochondria. Induction of autophagy has pro-survival effects through inhibiting apoptosis [5]. Short-term IHR induces preferential atrophy in the mouse diaphragm together with increased autophagy and enhanced fatigue resistance [6]. In contrast, long-term IHR-induced autophagy impairment induces cardiac hypertrophy, cardiac contractile dysfunction and myocardial injury through the 5' adenosine monophosphate-activated protein kinase pathway [7, 8]. Moreover, IHR preconditioning can aggravate the nerve injury of the global cerebral ischemia–reperfusion through the activation of mTOR/autophagy pathway [9]. Recent studies have shown that mesenchymal stem cell (MSC)-derived exosomes could reduce myocardial ischemia/reperfusion injury and diabetic nephropathy by inducing autophagy via AMPK/mTOR and Akt/mTOR pathways [10, 11]. In addition, bone marrow MSCs could mitigate the damage associated with ischemia/reperfusion injury, and silicosis through modulating activation of autophagy [12–14].

DNA methylation is both inheritable and reversible, and associated with a number of key processes including genomic imprinting, aging, and carcinogenesis. In mammalians, DNA methylation occurs most frequently on the 5’ carbon position of the pyrimidine ring of the cytosine residues in the sequence context of cytosine followed by guanine (CpG). About 40% of mammalian genes contain methylated CpG islands in their promoter and first exonic regions, which cause transcriptional silencing of genes. In contrast, DNA methylation in gene body regions relaxes chromatin structure, increasing gene expression [15]. Using whole genome DNA methylation analysis, we have identified *interleukin 1 receptor type 2* hypomethylation and *androgen receptor* hypermethylation as an important determinant of disease severity of OSA, and *natriuretic peptide 2* hypomethylation and *speckled protein 140* hypermethylation as a biomarker for vulnerability to excessive daytime sleepiness (EDS) in OSA [16]. Previous studies have found the relationship between aging and impaired autophagy as a result of increased DNA methylation of the autophagy related 5 (*ATG5*) and microtubule-associated protein 1 light chain 3 beta (*MAP1LC3B* or *LC3B*) genes, likely mediated by the DNA methyltransferase 3A and 3B [17, 18].

Little is known about the dynamic and functional role of autophagy activity and its epigenetic regulations in the development of OSA. We hypothesized that protein/gene expressions of the ATG5 (marker of autophagosome formation), LC3B (marker of autophagosome and lysosome fusion), and sequestosome 1 (SQSTM1 or p62, marker of autolysosome degradation), as well as DNA methylation levels of their gene promoter regions may be different in blood leukocytes from OSA patients and primary snoring (PS) subjects. In this prospective cohort study, we checked these autophagy-related markers in 64 OSA patients and 24 PS subjects in relation to clinical phenotypes. The development of atherosclerotic lesions results from a dynamic interplay between the native cells of the vasculature and different pro-inflammatory leukocytes issued from the general circulation, while leukocyte samples are readily accessible in OSA patients. Thus, we used leukocytes to measure autophagy-related markers. Furthermore, we checked these markers in human monocyte/endothelial cell culture models under IHR stimuli, and test their reversibility by cord-derived MSC, autophagy enhancer, and de-methylation/re-methylation agents in vitro.

## Methods

### Subjects

This study was approved by the Institutional Review Board of Chang Gung Memorial Hospital, Taiwan (Certificate number: 201802210B0). The participants were recruited from the sleep center and health examination center of Kaohsiung Chang Gung Memorial Hospital from July 2018 through June 2022. The informed consent was obtained from each subject participating in the study. The adults (aged 20–70 years) who were diagnosed as subject with PS (defined as apnea–hypopnea index (AHI) < 5) or OSA (defined as AHI ≥ 5) after the full night polysomnographic studies in our sleep laboratory were included. The exclusion criteria were ongoing infections, autoimmune disease, use of immunosuppressive agent in the past 6 months, narcolepsy, severe obesity (body mass index [BMI] ≥ 35 kg/m2), and those with a BMI < 21 kg/m2. The patients with OSA and PS were further divided into two subgroups according to EDS (Epworth sleepiness scale (ESS) of more than 10), cognitive dysfunction (Mail-In Cognitive Function Screening Instrument (MCFSI) scale of more than 4), depression (defined as at least 1 of the 2 core symptoms of low mood and loss of interest, or taking antidepressant medication at Psychiatric Clinic), or the presence of subjective daytime fatigue, subjective memory impairment, morning headache, or nocturia (> 2 events/night).

### Polysomnography

The complete polysomnography examination included electroencephalography, electrooculography, chin and anterior tibial electromyography, respiratory effort detectors, nasal/oral flow sensors, and pulse oximetry and was performed using a standardized commercial device (Sandman SD32 + TM Digital Amplifier [Embla, Colorado, U.S.A.)). All subjects completed their polysomnographic study with at least 4 h of total sleep time as indicated by electroencephalography. Sleep stage scoring was done by experienced technicians, and sleep parameters were defined according to the standard criteria [19].

### Measurements of ATG5, LC3B, and p62 protein expressions in monocytes and neutrophils by flowcytometry

To characterize the immune cell architecture in whole blood, a panel consisting of unstained cells, isotype control (mouse IgG conjugated to fluorescein isothiocyanate (FITC) and phycoerythrin (PE)), monocyte marker (CD14-PE-CyTM7, BD Pharmingen), neutrophil marker (CD16-PC5, Beckman Coulter), cytotoxic T cell marker (CD3-PE-Cy5; CD8-PE-Cy7), and helper T cell marker (CD3-PE-CyTM5, BD Pharmingen; CD4-PC7, Beckman Coulter) were chosen. To measure ATG5, LC3B, and p62 expressions in peripheral blood immune cells separately, APG5L/ATG5-Alexa Fluor 488 (abcam; USA), Anti-LC3B FITC (Millipore; USA), and Alexa Fluor®488 Mouse Anti-Human antibody for p62 (Millipore; USA) were used in combination with immune cell markers. After the lysis of erythrocytes with IntraPrep™ (Beckman Coulter; France), acquisition was performed on a CytomicsTM FC500 (Beckman Coulter; USA) using a dual staining protocol. The 5 × 10^4^ events were collected with immune cells gated in a side scatter versus frontal scatter plot. These were further analyzed for expressions of ATG5, LC3B, and p62 in CD16^+^ neutrophil, CD14^+^ monocyte, CD3^+^CD4^+^ helper T cell, and CD3^+^CD8^+^ cytotoxic T cell in the FL1 and FL2 channels, respectively. Analysis was performed using CXP Analysis software. Data were presented as percentage of stain positive cells or mean fluorescence intensity (MFI), which was corrected for background fluorescence with the corresponding isotype controls.

### Measuring ATG gene expressions in peripheral blood mononuclear cells (PBMCs) by quantitative real-time reverse transcription (RT)–polymerase chain reaction (PCR) method

PBMCs were isolated from heparinized blood of all study subjects using a two-layer Ficoll–Histopaque density gradient centrifugation (Histopaque 1.077 and 1.119; Sigma Diagnostics, St.Louis, MO) method. Total RNA from PBMCs was isolated by RNA Extraction RiboPureTM–Blood (Ambion), and converted to single stranded cDNA using a cDNA archive kit (Applied Biosystems) followed by the amplification of the ATG transcripts, including *LC3B*, *ATG5*, *p62*, *beclin 1 (BECN1*), *ATG9A*, and *unc-51-like autophagy activating kinase 1* (*ULK1*), using Taqman probe. Specific primers of *Glyceraldehyde 3-phosphate dehydrogenase* (*GAPDH*) was used as the internal control. Additional file 1: Table S1 lists the primer sequences. Relative expression levels were calculated using the 2^–∆∆Ct^ method with the median value for the control group as the calibrator. All the quantification was carried out in one go.

### Measuring CpG-site-specific DNA methylation levels by bisulfite pyro-sequencing method in the validation cohort

Genomic DNAs were extracted from PBMCs and bisulfite converted using the EZ DNA methylation kit from Zymo Research (CA, USA). PCR amplification primers and sequencing primers were designed by PyroMark Assay Design Software 2.0. PCR amplification of target region was performed using PyroMark PCR Kit (Qiagen). Seven promoter regions of the *LC3B*, *ATG5*, and *p62* genes were amplified based on reference sequence information from NCBI (*LC3B*: NM_022818.5; *ATG5*: NM_004849.4; *p62*: NM_003933.5). Bisulfite treatment was performed using an EpiTect 96 Bisulfite Kit (Qiagen) and PCR amplification was performed with a PyroMark PCR Kit (Qiagen). The primer sequences used for PCR amplification and pyro-sequencing for these regions are listed in Additional file 1: Table S1. The biotin-labeled PCR product was captured by Streptavidin-Sepharose HP (Amersham Pharmacia). Quantitation of cytosine methylation was performed using a PyroMark Q24 system (Qiagen). The amount of C relative to the sum of the amounts of C and T at each CpG site was calculated as a percentage [25]. Representative pyrograms of CpG di-nucleotides assayed of the *LC3B, ATG5,* and *p62* genes are presented in Additional file 1: Figures S1, S2, and S3.

### In vitro* human monocyte and endothelial cell line cultures under IHR circumference*

Human monocytic THP-1 cells or human umbilical vein endothelial cells (HUVEC) were added into 24-well plates (adjusted to 1 × 10^6^ cells per ml), and then were exposed to normoxia (NOX) or IHR in a custom-designed, incubation chambers which are attached to an external O2–CO2–N2 hand-driven controller. Air-phase set point consisted of a 25-min hypoxic period (0% O2 and 5% CO2), followed by 35 min of re-oxygenation (21% O2 and 5% CO2), 7 h each day for 9 days. Protein expression levels of the ATG5, LC3B, and p62 in THP-1 cells were determined using flowcytometry method, as described above.

### Measurements of ROS production by 2′,7′-Dichlorodihydrofluorescein diacetate (H2DCFDA)

0.1 μM of working solution of fluorochrome marker H2DCFDA (catalog no. D6883; Sigma, USA) was added to the THP-1 or HUVEC cells, which were submit to flow cytometry on a CytomicsTM FC500 (Beckman Coulter; USA) for ROS detection using the 488 nm laser for excitation and detected at 535 nm.

### Isolation and culture of human umbilical cord MSCs

Primary human umbilical cord MSCs were isolated from the human umbilical cord of a human subject. It was approved by the ethics committee of Kaohsiung Chang Gung Memorial Hospital and conformed to the regulatory standards. Umbilical cord tissue was harvested from the obtained human umbilical cord soaked in HBSS Solution (Cat. 14025–092, Gibco) within 24 h. Briefly, the tissue pieces were put into a dish containing Wharton Jelly culture medium and incubated at 37 °C and 5% CO2 for about 7 days to let the mesenchymal stem cells move into medium from the umbilical cord. MSCs (3 × 10^4^ cells) were plated in 24-well plates for 24 h to produce MSC-conditioned medium (CM).

### Statistical analysis

Data were expressed as the mean ± standard deviation. Independent Student *T* test Kruskal–Wallis H, or Mann–Whitney *U* test was used for comparing continuous values of different groups where appropriate. Categorical variables were analyzed using Chi-square test. Pearson’s correlation was used to determine the relationship between selected variables. Stepwise multiple linear regression analysis with all potential co-variables, including age, sex, BMI, AHI, ESS, history of smoking, history of alcoholism, and co-morbidities, entered in a single step was used to adjust p values in the comparisons of ATG protein/gene expression and DNA methylation levels. A *p* value of less than 0.05 is considered statistically significant.

## Results

A total of 24 PS and 64 patients with treatment-naïve OSA were analyzed. There were no significant differences between two groups in terms of age, sex, BMI, smoking history, co-morbidity, blood lipid profiles, and fasting blood sugar, but significant differences in sleep parameters. In addition, OSA patients had higher ESS and MCFSI, and had larger proportions of noctuia and subjective daytime fatigue versus PS subjects (Table 1).

**Table 1:** Demographic, biochemistry, and sleep data of all the 88 study participants in the validation cohort

	PS subjects (*n* = 24)	Severe OSA patients (*n* = 64)	*p* value
Age, years	45.6 ± 10.7	47 ± 11.1	0.595
Male sex, *n* (%)	17 (70.8)	53 (82.8)	0.215
BMI, kg/m^2^	25 ± 3.5	25.8 ± 2.7	0.259
AHI, events/hour	4.2 ± 3	47.1. ± 19.4	< 0.001
ODI, events/hour	1.1 ± 0.9	33.0 ± 23.5	< 0.001
Mean SpO2, %	94.8 ± 1.5	92.5 ± 12.7	0.606
Minimum SpO2, %	90.0 ± 2.2	75.5 ± 17.6	0.024
Snoring index, counts/hour	139 ± 126	390 ± 214	0.008
Arousal index, events/hour	21.9 ± 18.1	48.7 ± 30.9	0.014
Epworth sleepiness scale	5.5 ± 4.1	9.5 ± 4.2	0.032
Excessive daytime sleepiness, *n* (%)	4 (16.7)	25 (39.1)	0.089
MCFSI	1.917 ± 2.376	3.305 ± 2.571	0.024
Cognitive dysfunction, *n* (%)	4 (16.7)	17 (26.6)	0.332
Daytime fatigue, *n* (%)	8 (33.3)	44 (68.8)	0.007
Memory impairment, *n* (%)	12 (50)	40 (42.5)	0.546
Morning headache, *n* (%)	4 (16.7)	14 (21.9)	0.759
Nocturia, *n* (%)	7 (29.2)	37 (57.8)	0.044
Smoking history, *n* (%)	10 (41.7)	21 (32.9)	0.625
Alcoholism history, *n* (%)	0 (0)	1 (1.6)	0.538
Cholesterol, mg/dl	192.4 ± 31.2	195.5 ± 37.9	0.95
Triglycerides, mg/dl	129.1 ± 80.5	149.1 ± 132.3	0.497
Co-morbidities			
Hypertension, *n* (%)	6 (25)	21 (32.8)	0.479
Diabetes mellitus, *n* (%)	3 (12.5)	4 (6.3)	0.335
Heart disease, *n* (%)	1 (4.2)	5 (7.8)	0.546
Stroke, *n* (%)	0 (0)	2 (3.1)	0.381
CKD, *n* (%)	1 (4.2)	1 (1.6)	0.465
Depression, *n* (%)	2 (8.3)	16 (25)	0.125

*PS* Primary snoring; *HS* Healthy subjects; *BMI* Body mass index; *AHI* Apnea hypopnea index; *ODI* Oxygen desaturation index; *SpO2* Pulse oxyhemoglobin saturation; *ESS* Epworth Sleepiness Scale; *EDS* Excessive daytime sleepiness; *MCFSI* Mail-in cognitive function screening instrument; *CKD* Chronic kidney disease

### Decreased LC3B protein expressions of blood monocyte, decreased ATG5 protein expressions of blood neutrophil, and increased p62 protein expressions of blood cytotoxic T cell in OSA patients versus PS

**Fig. 1:**
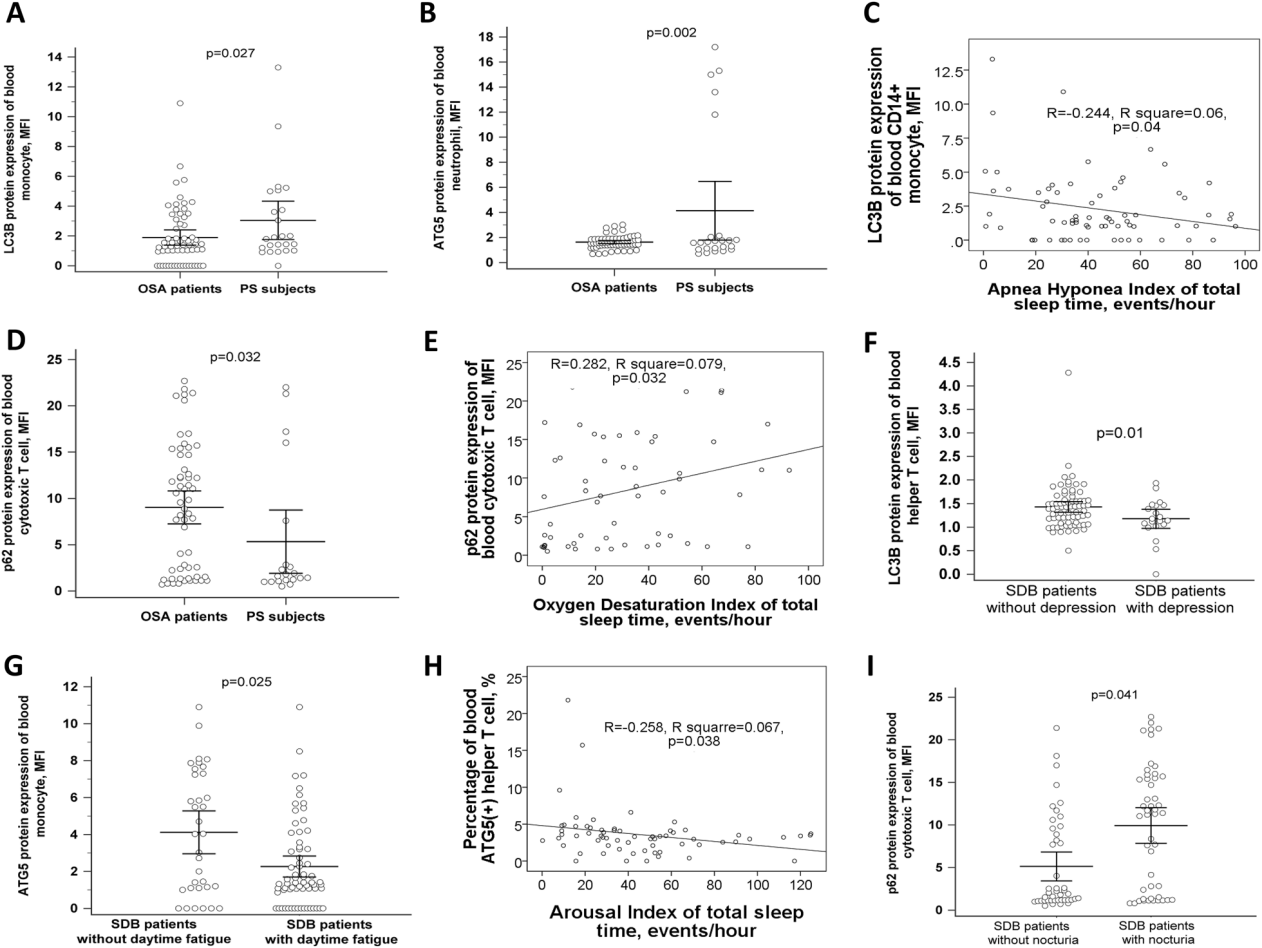
Impaired autophagy flux in patients with obstructive sleep apnea (OSA) and those with specific phenotypes. **A** LC3B protein expression of blood monocyte was decreased in OSA patients versus subjects with primary snoring (PS). **B** ATG5 protein expression of blood neutrophil was decreased in OSA patients versus PS subjects. **C** LC3B protein expression of blood monocyte was negatively correlated with apnea hyponea index (AHI). **D** P62 protein expression of blood cytotoxic T cell was increased in OSA patients versus PS subjects, and **E** positively correlated with oxygen desaturation index (ODI). **F** LC3B protein expression of blood helper T cell was decreased in sleep disordered breathing (SDB) patients with depression versus those without depression. **G** ATG5 protein expression of blood monocyte was decreased in SDB patients with subjective daytime fatigue versus those without fatigue. **H** Percentage of blood ATG5 ( +) helper T cell was negatively correlated with arousal index of total sleep time. **I** P62 protein expression of blood cytotoxic T cell was increased in SDB patients with nocturia versus those with nocturia

**Fig. 2:**
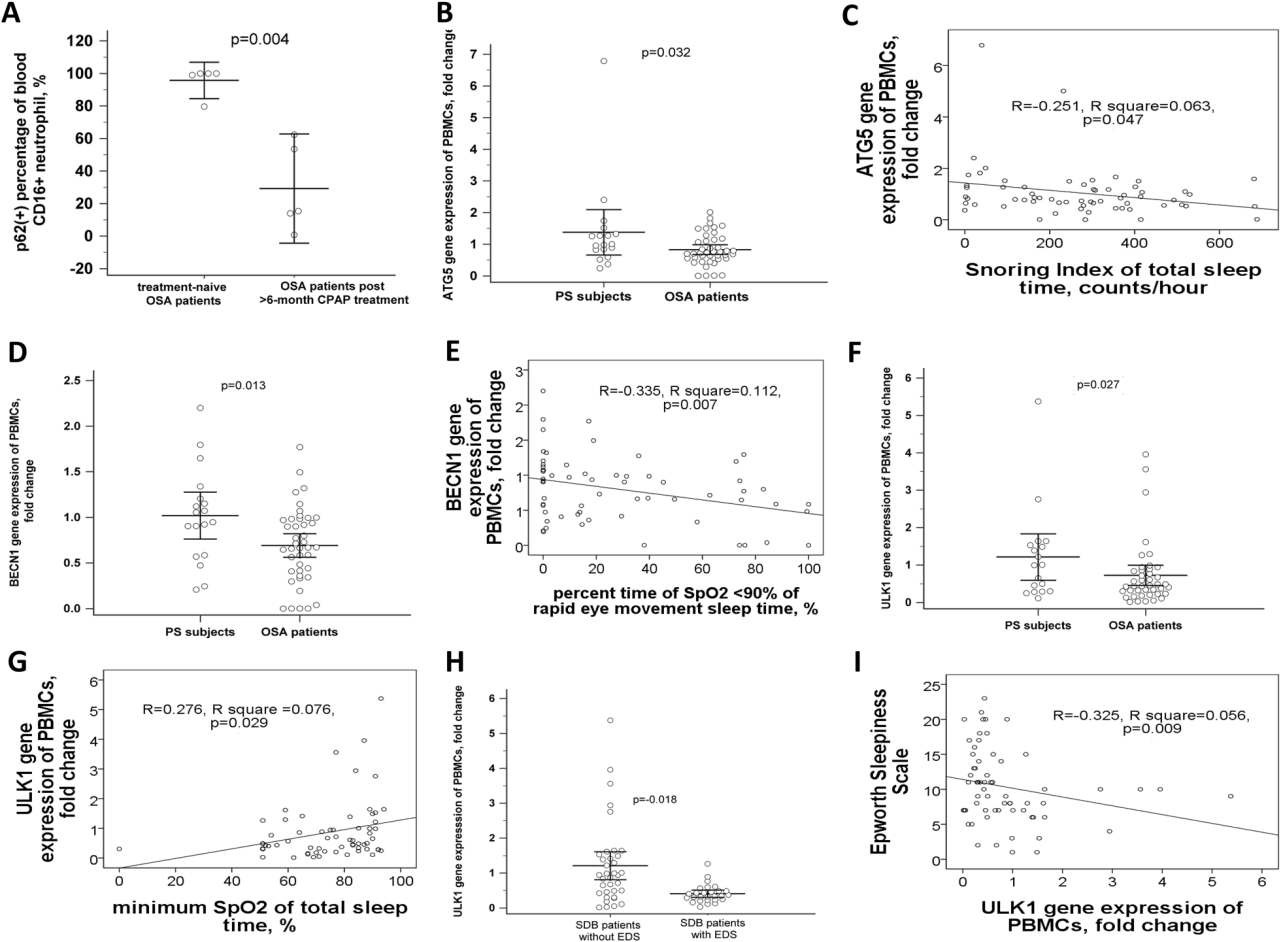
Decreased gene expressions of the autophagy-related genes (ATG) in OSA patients and those with excessive daytime sleepiness. **A** Percentage of blood p62( +) neutrophil was reduced after > 6-month continuous positive airway pressure treatment at home in five OSA patients. **B** *ATG5* gene expression of peripheral blood mononuclear cells (PBMCs) was decreased in OSA patients versus PS subjects, and **C** negatively correlated with snoring index of total sleep time. **D** *BECN1* gene expression of PBMCs was decreased in OSA patients versus PS subjects, and **E** negatively correlated with percent time of SpO2 less than 90% during rapid eye movement sleep. **F** *ULK1* gene expression of PBMCs was decreased in OSA patients versus PS subjects, and **G** positively correlated with minimum SpO2 of total sleep time. **H** *ULK1* gene expression was further decreased in sleep disordered breathing (SDB) patients with excessive daytime sleepiness (EDS) versus those without EDS, and **I** negatively correlated with Epworth Sleepiness Scale

Both LC3B protein expression of peripheral blood CD14^+^ monocyte (1.89 ± 2.01 versus 3.04 ± 3.04 MFI, adjusted *p* = 0.027, Fig. 1A) and ATG5 protein expression of blood CD16^+^ neutrophil (1.63 ± 0.5 versus 4.13 ± 5.56 MFI, adjusted *P* = 0.002, Fig. 1B) were decreased in OSA patients as compared with that in PS subjects, and the former was negatively correlated with AHI (*R* = − 0.244, *p* = 0.04, Fig. 1C). P62 protein expression of blood CD4^+^CD8^+^ cytotoxic T cell (9.04 ± 6.81 versus 5.33 ± 7.33, adjusted *p* = 0.032, Fig. 1D) was increased in OSA patients as compared with that in PS subjects, and positively correlated with ODI (*R* = 0.282, *p* = 0.032, Fig. 1E). Subgroup analysis showed that LC3B protein expression of blood CD3^+^CD4^+^ T helper cell was further decreased in sleep-disordered breathing (SDB) patients with depression as compared with that in those without depression (1.18 ± 0.43 versus 1.43 ± 0.48, adjusted *p* = 0.01, Fig. 1F). ATG5 protein expression of blood monocyte was decreased in SDB patients with daytime fatigue versus those without daytime fatigue (2.26 ± 2.36 versus 4.12 ± 3.33, adjusted *p* = 0.025, Fig. 1G), and ATG5( +) percentage of blood CD3^+^CD4^+^ helper T cell was negatively correlated with arousal index (*R* = − 0.258, *p* = 0.038, Fig. 1H). P62 protein expression of blood cytotoxic T cell (9.93 ± 7.2 versus 5.12 ± 5.61 MFI, adjusted *p* = 0.041, Fig. 1I) was increased in SDB patients with nocturia versus those without nocturia. In five OSA patients whose blood samples were obtained again after more than 6-month CPAP treatment, p62( +) percentage of blood CD16^+^ neutrophils was reduced (29.2 ± 27% versus 95.7 ± 9%, *p* = 0.004, Fig. 2A), while none of the other parameters showed normalization after CPAP treatment.

### Decreased gene expressions of the ATG5, BECN1, and ULK1 genes in OSA patients versus PS subjects, and further decreased ULK1 gene expression in those with EDS

Gene expression levels of the 6 selected ATGs, including *ATG5, BECN1, ULK1, ATG9A, P62,* and *LC3B*, were measured in the PBMC samples. *ATG5* gene expression was deceased in OSA patients versus PS subjects (0.83 ± 0.49 versus 1.38 ± 1.44 fold change, adjusted *p* = 0.032, Fig. 2B), and negatively correlated with snoring index (*R* = 0.251, *p* = 0.047, Fig. 2C). *BECN1* gene expression was decreased in OSA patients versus PS subjects (0.69 ± 0.41 versus 1.02 ± 0.52 fold change, adjusted *p* = 0.013, Fig. 2D), and negatively correlated with percent time of SpO2<90% during rapid eye movement sleep (*R* = − 0.335, *p* = 0.007, Fig. 2E). *ULK1* gene expression was decreased in OSA patients versus PS subjects (0.73 ± 0.86 versus 1.22 ± 1.25 fold change, adjusted *p* = 0.027, Fig. 2F), and positively correlated with minimum SaO2 during total sleep time (*R* = 0.276, *p* = 0.029, Fig. 2G). *ULK1* was further down regulated in those with EDS (0.43 ± 0.28 versus 1.02 ± 1.13 fold change, adjusted *p* = 0.018, Fig. 2H), and negatively correlated with ESS (*R* = − 0.325, *p* = 0.009, Fig. 2I).

### Increased DNA methylation levels of the LC3B gene promoter regions in OSA patients, particularly in those with EDS

**Fig. 3:**
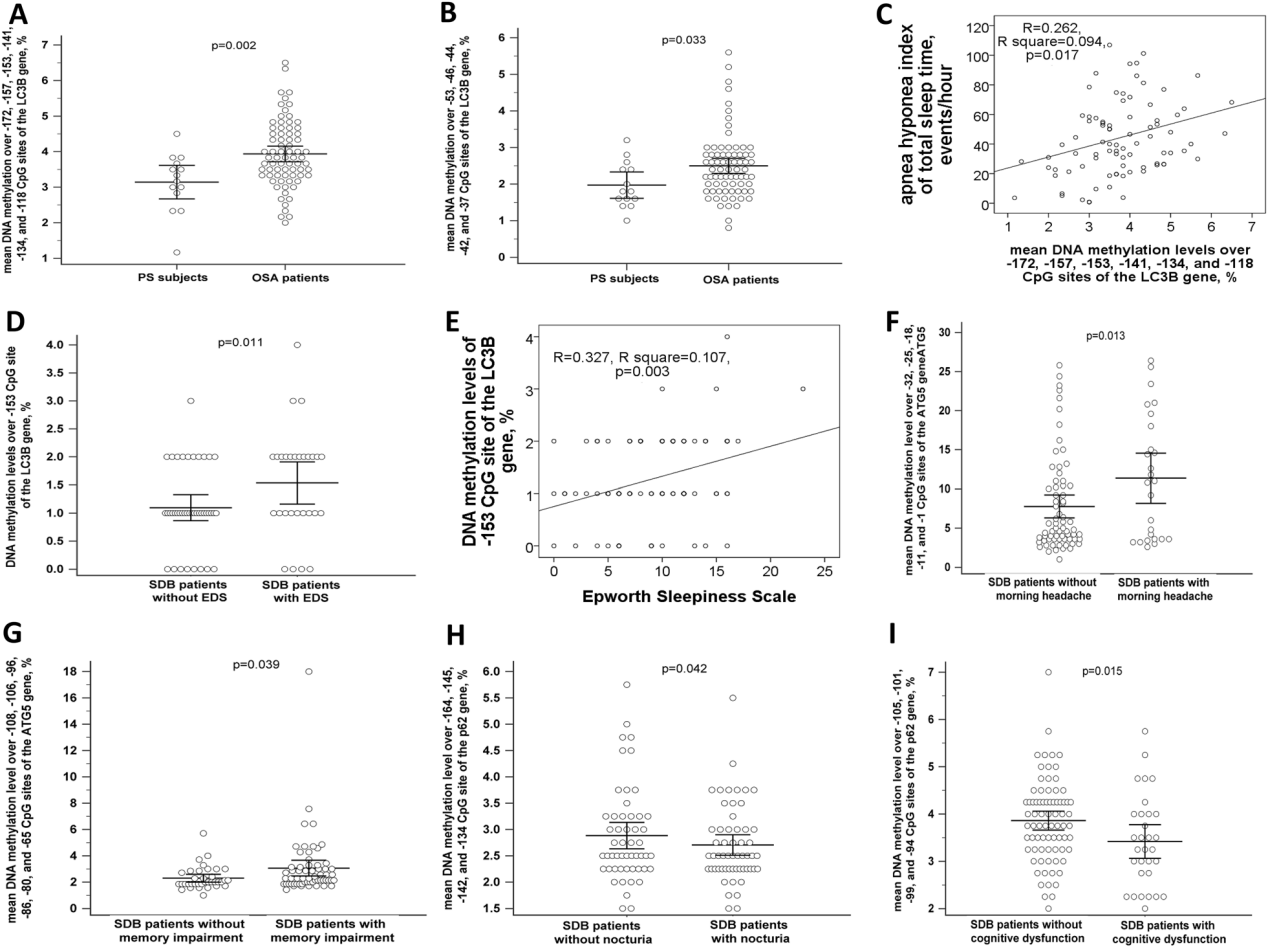
Aberrant DNA methylation patterns of the ATG genes in OSA patients and those with specific phenotypes. **A** Mean DNA methylation levels over − 172, − 157, − 153, − 141, − 134, and − 118 CpG sites of the *LC3B* gene were increased in OSA patients versus PS subjects. **B** Mean DNA methylation levels over − 53, − 46, − 44, − 42, and − 37 CpG sites of the *LC3B* gene were increased in OSA patients versus PS subjects. **C** Mean DNA methylation levels over − 172, − 157, − 153, − 141, − 134, and − 118 CpG sites of the *LC3B* gene were positively correlated with apnea hyponea index. **D** DNA methylation levels over -153 CpG site of the *LC3B* gene were further increased in SDB patients with EDS versus those without EDS, and **E** positively correlated with Epworth Sleepiness Scale. **F** Mean DNA methylation levels over − 32, − 25, − 18, − 11 and − 1 CpG sites of the *ATG5* gene were increased in SDB patients with morning headache versus those without headache. **G** Mean DNA methylation levels over − 108, − 106, − 96, − 86, − 80, − 65, and − 61 CpG sites of the *ATG5* gene were increased in SDB patients with subjective memory impairment versus those with memory impairment. **H** Mean DNA methylation levels over − 164, − 145, − 142, and − 134 CpG sites of the *p62* gene were decreased in SDB patients with nocturia versus those without nocturia. **I** Mean DNA methylation levels over − 105, − 101, − 99, and − 94 CpG sites of the *p62* gene were decreased in SDB patients with cognitive dysfunction versus those without cognitive dysfunction

Mean DNA methylation levels over − 172, − 157, − 153, − 141, − 134, and − 118 CpG sites of the *LC3B* gene (3.93 ± 0.94 versus 3.24 ± 0.82%, adjusted *p* = 0.002, Fig. 3A) and mean DNA methylation levels over − 53, − 46, − 44, − 42, and − 37 CpG sites of the *LC3B* gene (2.5 ± 0.89 versus 1.97 ± 0.62%, adjusted *p* = 0.033, Fig. 3B) were both increased in OSA patients versus PS subjects, and the former was positive correlated with AHI (*R* = 0.262, *p* = 0.017, Fig. 3C). In addition, mean DNA methylation levels over − 97, − 90, − 80, − 67, − 63, − 53, − 46, − 44, − 42, and − 37 CpG sites of the *LC3B* gene were also positively correlated with AHI (*R* = 0.299, *p* = 0.002). Subgroup analysis showed that DNA methylation level over − 153 CpG site of the *LC3B* gene was further increased in SDB patients with EDS versus those without EDS (1.54 ± 0.96 versus 1.1 ± 0.74, adjusted *p* = 0.011, Fig. 3D), and was positively correlated with ESS (*R* = 0.327, *p* = 0.003, Fig. 3E). Mean DNA methylation levels over − 32, − 25, − 18, − 11 and − 1 CpG sites of the *ATG5* gene were increased in SDB patients with morning headache versus those without morning headache (11.36 ± 7.88 versus 7.77 ± 6.11%, adjusted *p* = 0.013, Fig. 3F). Mean DNA methylation levels over − 108, − 106, − 96, − 86, − 80, − 65, and − 61 CpG sites of the *ATG5* gene were increased in SDB patients with memory impairment versus those without memory impairment (3.07 ± 2.33 versus 2.3 ± 0.87%, adjusted *p* = 0.039, Fig. 3G). Mean DNA methylation levels over − 164, − 145, − 142, and − 134 CpG sites of the *p62* gene were decreased in SDB patient with nocturia versus those without nocturia (2.74 ± 0.33 versus 3.05 ± 0.91%, adjusted *p* = 0.042, Fig. 3H). Mean DNA methylation levels over − 105, − 101, − 99, and − 94 CpG sites of the *p62* gene were decreased in SDB patients with cognitive dysfunction versus those without cognitive dysfunction (3.56 ± 0.99 versus 4.01 ± 0.83%, adjusted *p* = 0.015, Fig. 3I).

### Decreased autophagy flux, increased ROS production, and increased late apoptosis in THP-1 and HUVEC cells in response to IHR stimuli, and their reversions with either rapamycin (autophagy enhancer) or MSC treatment via de-methylation of the ATG5 promoter region

**Fig. 4:**
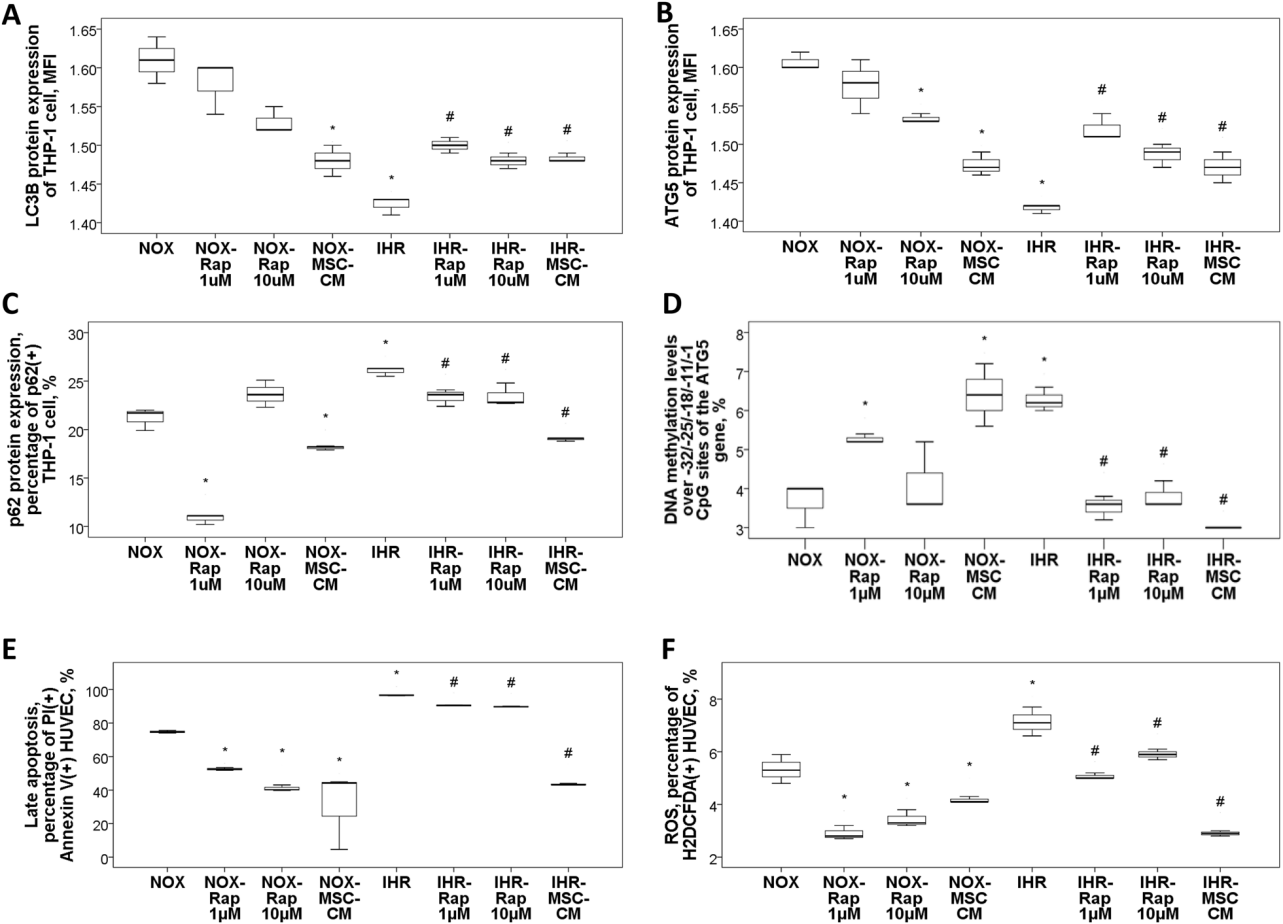
Autophagy enhancer and mesenchymal stem cell (MSC) treatment reversed intermittent hypoxia with re-oxygenation (IHR)-induced autophagy impairment, late apoptosis and oxidative stress. Either rapamycin (Rap) or human umbilical cord-derived MSC-condition medium (CM) reversed IHR-induced decreased **A** LC3B and **B** ATG5 protein expressions, and **C** increased p62 protein expression in THP-1 cells. **D** Rap and MSC-CM also reversed IHR-induced hypermethylation over − 32, − 25, − 18, − 11, and − 1 CpG sites of the *ATG5* gene. Either Rap or MSC-CM reversed IHR-induced **E** late apoptosis and **F** over-production of reactive oxygen species (ROS) in HUVEC *p < 0.05, compared between IHR and normoxic (NOX) condition #*p* < 0.05, compared between IHR plus specific treatment and IHR alone condition

Late apoptosis (percentage of Annexin V ( +) and PI( +)) of THP-1 cells was increased with 9-day IHR stimuli, and partly reversed with either rapamycin or MSC-CM treatment (Additional file 1: Fig. S4). LC3B (Fig. 4A) and ATG5 (Fig. 4B) protein expressions of THP-1 cells were both decreased in response to IHR stimuli versus NOX condition, and p62 (Fig. 4C) protein expression was increased, while they were all reversed with either rapamycin or MSC-CM treatment.

Gene expression levels of the *LC3B*, *ATG5*, *ATG9A*, and *BECN1* genes in THP-1 cells were all decreased in response to IHR stimuli versus NOX condition, while *p62* gene expression was increased (Additional file 1: Fig. S4). Treatment with either rapamycin (1 or 10 μM) or 50% MSC-CM under IHR condition reversed gene expression levels of these five ATGs versus IHR alone condition. In addition, mean DNA methylation levels over − 32/− 25/− 18/− 11/− 1 CpG sites of the *ATG5* gene were increased with IHR stimuli, and decreased with either Rap or MSC-CM treatment versus IHR alone condition (Fig. 4D). In contrast, no significant change in DNA methylation levels of the *LC3B* or *P62* gene promoter regions in response to IHR stimuli was noted. Moreover, late apoptosis (Fig. 4E) and ROS production (Fig. 4F) of HUVEC were both increased with IHR stimuli versus NOX condition, and significantly reversed with either rapamycin or MSC-CM treatment.

### De-methylation agent (5-Aza-2′-deoxycytidine; 5-AZA) and re-methylation agent (folic acid; FA) reversed IHR-induced oxidative stress and autophagy impairment in THP-1 cells

**Fig. 5:**
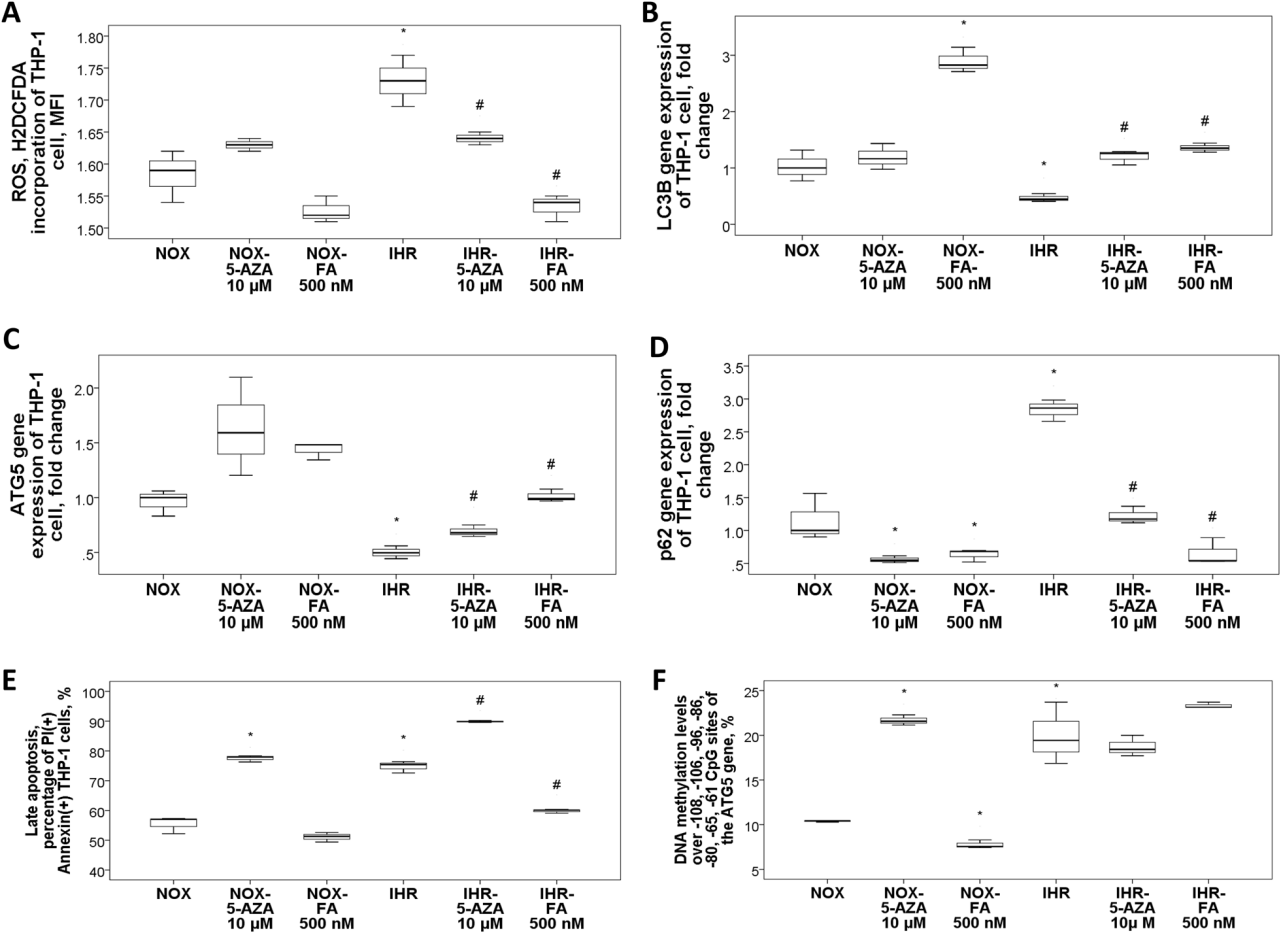
De-methylation and re-methylation agents reversed IHR-induced late apoptosis and autophagy impairment. Either de-methylation agent (5-Aza-2′-deoxycytidine; 5-AZA) or re-methylation agent (Folic Acid; FA) reversed IHR-induced **A** over-production of reactive oxygen species (ROS), as well as down-regulations of **B** *LC3B* and **C** *ATG5* genes and **D** *p62* up-regulation. **E** Only FA reversed IHR-induced late apoptosis. **F** Mean DNA methylation levels over − 108, − 106, − 96, − 86, − 80, − 65, and − 61 CpG sites of the *ATG5* gene were increased with IHR stimuli versus normoxic (NOX) condition, but remained unchanged with either 5-AZA or FA treatment under IHR condition. *p < 0.05, compared between IHR and normoxic (NOX) condition #*p* < 0.05, compared between IHR plus specific treatment and IHR alone condition

Both 5-AZA and FA reversed IHR-induced over production of ROS (Fig. 5A), and reversed IHR-induced *LC3B/ATG5* down-regulation and *p62* up-regulation (Fig. 5B–D), while only FA reversed IHR-induced late apoptosis (Fig. 5E). Mean DNA methylation levels over − 108, − 106, − 96, − 86, − 80, − 65, and − 61 CpG sites of the *ATG*5 gene was increased with IHR versus NOX condition, but remained the same with either 5-AZA or FA treatment (Fig. 5F).

## Discussion

In the current study, we found that OSA patients had impaired autophagy activity (decreased LC3B/ATG5/BECN1/ULK1 expressions, and increased p62 accumulation) and increased DNA methylation over promoter regions of the *LC3B* gene as compared with PS subjects. OSA patients with neuropsychiatric symptoms, such as depression, fatigue, sleepiness, headache, and memory impairment, had higher LC3B/ATG5 expression and lower DNA methylation levels over the promoter regions of these two genes. In contrast, OSA patients with nocturia had lower p62 expression and higher DNA methylation levels at the *p62* gene promoter region. In addition, we provided evidence that in vitro 9-day IHR program resulted in autophagy impairment (decreased LC3B/ATG5 expression and increased p62 expression) along with increased DNA methylation levels at the *ATG5* gene promoter region, increased apoptosis, and increased oxidative stress, which were reversed with rapamycin, MSC, or FA treatment. Thus, clinical phenotypes of OSA may be triggered by chronic IHR-induced autophagy impairment through epigenetic regulations.

Autophagy has been considered to be a double-edged sword, protective or deteriorative, for neuronal and endothelial survival after cerebral ischemia or persistent hypoxia depending on the degree and duration of the condition [20, 21]. Several animal models have demonstrated that chronic intermittent hypoxia induced cardiac hypertrophy, nerve injury, and endothelial injury by impairing autophagy, whereas others showed that intermittent hypoxia-induced autophagy activation attenuates cardiac contractile dysfunction, pancreatic beta-cell apoptosis, sensory nerve dysfunction, or renal tubular epithelial cell injury [7–9, 22–29]. For the first time, we found that autophagy activity was impaired in OSA patients, particularly in those with neuropsychiatric symptoms. In line with our findings, a recent study showed that treatment with OSA patients-derived exosomes decreased the expression of the autophagy markers LC3B II/I and beclin1 [30]. We further demonstrated the effects of long-term 9-day IHR on autophagy impairment, oxidative stress, and late apoptosis in vitro, and verified the reversibility with autophagy enhancer or MSC treatment. Notably, the effects of varying degrees and duration of IHR on autophagy in hippocampal neurons are different [31]. Short-term IHR-mediated autophagy activation is able to maintain organ functions by attenuating tissue necrosis, whereas OSA is characterized by chronic intermittent hypoxia and may not be mimicked by the IHR protocols of too short duration. Recently, there is accumulating evidence linking the impairment of the autophagy–lysosomal pathway with neurodegenerative disorder, such as Alzheimer's disease and Parkinson's disease [32, 33], as well as in progressive kidney diseases [34]. Recent research has validated autophagy decline as one of the central pathways in the promotion of functional loss and increased vulnerability to disease associated with aging [35]. Further studies are required to clarify underlying mechanisms by which chronic IHR in OSA leads to autophagy impairment and cell injury.

To date, only a few autophagy-related genes and signaling molecules have been reported to undergo DNA methylation and thus affect autophagy activity or contribute to autophagy-related diseases. A recent animal study found that maternal e-cigarette exposure down regulated autophagy-related gene expression via DNA hypermethylation of the *ATG5* gene promoter, leading to programming of a hypoxic–ischemic sensitive phenotype in the neonatal brain [36]. Another study reported that de-methylation agent, 5-AZA, increased LC3B levels, autophagy activity, and cell apoptosis in human chronic myeloid leukaemia cell lines [37]. In the current study, we found that DNA methylation levels of the *LC3B* gene promoter regions were increased in OSA patients, particularly in those with EDS, and positively correlated with AHI. Hypermethylation of the *ATG5* gene promoter region and hypomethylation of the *p62* gene promoter region were noted in OSA patients with neurocognitive dysfunction and nocturia, respectively. However, in vitro 9-day IHR stimuli did not resulted in any change in DNA methylation levels of these ATG genes, except for hypermethylation over two promoter regions of the *ATG5* gene. Theoretically, inherited DNA methylation patterns (epigenotype) may affect the development of OSA, while chronic intermittent hypoxia stimuli may cause disease progression through DNA methylation changes. We speculate that hypermethylated *LC3B* gene promoter and hypomethylated *p62* gene promoter may be inheritable epigenotypes responsible for susceptibility to OSA and its adverse consequences through impairing autophagy activity, while hypermethylated *ATG5* gene promoter regions may contribute to morning headache or subjective memory impairment in OSA patients in response to chronic intermittent hypoxia exposure. Further studies are needed to clarify the cause and effect relationship between OSA and DNA methylation change of the ATG genes.

The selective autophagy receptor and ubiquitin sensor, p62, is localized to the autophagosome via LC3-interaction and is constantly degraded by the autophagy–lysosome system, so ablation of autophagy leads to marked accumulation of p62, which can promote ROS-induced DNA damage and cell death, and induce interleukin-1 and 6 expression via activating nuclear factor kappa B [38, 39]. DNA methylation and repressive H3K9me3 and H3K27me3 marks in the *p62* gene promoter can overcome the radioresistance of head and neck cancer cells via autophagy-dependent senescence induction [40]. In the current study, we found that OSA patients with nocturia had higher p62 protein expression in association with decreased DNA methylation levels over the *p62* promoter region. The pathophysiology explaining the relationship between OSA and nocturia remains poorly understood, and may involve several possible mechanisms, including hypoxemia, changes in intra-thoracic pressure, and changes in renin–angiotensin–aldosterone axis [41]. Our findings suggest that epigenetics-related p62 accumulation may play a role in the development of nocturia in OSA patients.

Several drugs have been proven to up regulate autophagy and may benefit health and lifespan [35]. In the current in vitro experiments, we demonstrated that either rapamycin or MSC reversed long-term IHR-induced autophagy impairment, oxidative stress, and cell apoptosis, partly through de-methylation of the *ATG5* gene promoter region. Abundant evidence demonstrates the beneficial impact of MSCs on cerebral ischemic injury and atherosclerosis through suppressing ROS generation, transferring healthy mitochondria to damaged cells, inhibiting pro-inflammatory cells activation, and regulating autophagy imbalances [42, 43]. On the other hand, we found that both 5-AZA and FA treatment reduced IHR-induced over-production of ROS and autophagy impairment, whereas only FA reversed IHR-induced late apoptosis, suggesting a potential role of the epigenetic drugs in the treatment of OSA-related adverse consequences. In line with this finding, FA has been shown to reverse IHR-induced oxidative stress and cell apoptosis through hypermehtylation of the *formyl peptide receptor* 1 gene promoter region [44]. Further studies are needed to identify specific ATG genes responsible for this benefit.

Several limitations in the current study should be acknowledged. First, we did not verify the DNA methylation data in a single type of cells. It has been shown that differences in monocyte and lymphocyte cell distributions may contribute to the observed variation in PBMC DNA methylation, and the adjustment for cell-type distributions or single cell DNA methylation analysis may help to minimize the confounding when analyzing blood-derived DNA methylation data, particularly in situations, where the disease or exposure of interest is responsible for shifts in leukocyte subpopulations [45, 46]. Second, in the current study, we did not measure mitochondrial mass or ROS levels in the blood samples, but only measured blood leukocyte macroautophagy flux, which involves the formation of cytosolic vesicles (autophagosomes) that seize the cargo, blend with the lysosome and release the contents within it. The selective autophagic degradation of mitochondria is called “mitophagy”, which is a mechanism supporting the integrity of mitochondrial networks following ROS formation under stress conditions to protect from the accumulation of mitochondrial DNA mutations. Further study is needed to clarify the role of mitophagy in OSA [47]. Third, we did not measure levels or activity of the enzymes that catalyze DNA methylation or de-methylation process. DNA methylation is catalyzed by DNA methyltransferases (DMNTs). DNMT1 is generally responsible for maintenance of methylation, while DNMT3a and DNMT3b perform de novo methylation. Hypoxia-mediated change in DNA methylation levels alters accessibility of genes for transcription factors and change the rates of gene transcription. Cyclic exposure to hypoxia up-regulates hypoxia inducible factor-1, and down-regulate superoxide dismutase 2 and endothelial nitric oxide synthase 3, through modulating DNA methylation levels of these gene promoter regions [48].

**Fig. 6:**
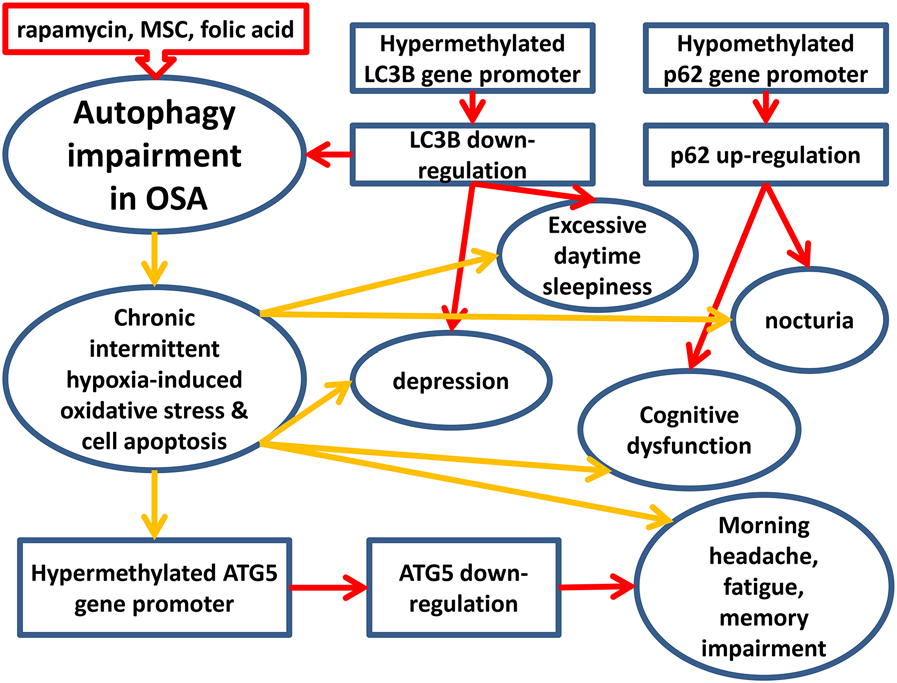
Proposed model of epigenetics-regulated autophagy impairment in obstructive sleep apnea (OSA) and its clinical phenotypes. A schematic diaphragm depicts probably contributing effects of hypermethylated *LC3B* gene promoters and hypomethylated *p62* gene promoters on the development of OSA and EDS/nocturia/depression/cognitive dysfunction phenotypes through inhibiting autophagy. In contrast, chronic intermittent hypoxia in OSA patients may lead to hypermethylated *ATG5* gene promoters, which probably contribute to the development of morning headache, memory impairment, and fatigue through down regulating ATG5. Autophagy enhancer (rapamycin), mesenchymal stem cell, and re-methylation agent (folic acid) treatment may reduce late apoptosis and oxidative stress through augmenting autophagy. Red arrows indicate predisposition, and orange arrows indicate cause and effect relationships

Based on the clinical data, we showed that OSA patients had decreased LC3B expression than PS subjects in association with hypermethylation of the *LC3B* gene promoter regions, while both markers were correlated with AHI. Moreover, OSA with neuropsychiatric or nocturia symptoms had further impaired autophagy in association with aberrant DNA methylation of the ATG gene promoter regions. In the basic experiments, we found that rapamycin, MSC, and FA could reverse long-term IHR-induced ROS over-production, late apoptosis, and autophagy impairment. Our findings provide initial evidence to support the potential effectiveness of autophagy enhancers for ameliorating the progression of OSA and preventing the occurrence of its adverse consequences. Autophagy may exert this influential protective effect on the development of OSA by acting on several molecular pathways via inherited epigenotypes of the *LC3B* and *p62* genes, while chronic IHR may lead to various adverse consequences through autophagy impairment via hypermethylation of the *ATG5* gene promoter regions (Fig. 6). Future studies are necessary to determine the hierarchic organization of this dual interplay of autophagy with OSA, and to identify specific DNA methylation patterns of the ATGs contributing to the benefit of the autophagy enhancers. Based on our findings, augmenting the autophagy-related pathways seems to be a promising tool for developing novel therapeutics for OSA, although findings point to the fact that the underlying molecular mechanisms must be defined before it can be effectively exploited in pharmaceutical areas.

## Supplementary Information

**Additional file 1: Table S1.** Primer sequences for assaying quantitative real-time polymerase chain reactions and pyro-sequencing used in the present study. **Figure S1.** Pyrograms of the representative CpG sites assayed of the *LC3B* gene promoter region. **Figure S2.** Pyrograms of the representative CpG sites assayed of the *ATG5 *gene. **Figure S3.** Pyrograms of the representative CpG sites assayed of the *p62* gene. **Figure S3.** Pyrograms of the representative CpG sites assayed of the *p62* gene. **Figure S4.** Effects of rapamycin (Rap) and mesenchymal stem cell (MSC) treatment on late apoptosis and autophagy related gene (ATG) expressions under intermittent hypoxia with re-oxygenation stimuli (IHR) in THP-1 cells.

## Data Availability

All data generated or analysed during this study are included in this published article and its Additional files.

## Abbreviations

OSA: Obstructive sleep apnea
IHR: Intermittent hypoxia with re-oxygenation
CPAP: Continuous positive airway pressure
ROS: Reactive oxygen species
MSC: Mesenchymal stem cell
CpG: Cytocine guanine
EDS: Excessive daytime sleepiness
ATG5: Autophagy related 5
LC3B: Microtubule-associated protein 1 light chain 3 beta
p62: Sequestosome 1
PS: Primary snoring
AHI: Apnea hyponea index
BMI: Body mass index
MCFSI: Mail-In Cognitive Function Screening Instrument
SpO2: Pulse oxygen saturation
MFI: Mean fluorescence intensity
PBMC: Peripheral blood mononuclear cell
RT-PCR: Reverse transcriptase polymerase chain reaction
BECN1: Beclin 1
ULK1: Unc-51 like autophagy activating kinase 1
HUVEC: Human umbilical vein endothelial cell
NOX: Normoxic
SDB: Sleep disordered breathing
5-AZA: 5-Aza-2′-deoxycytidine
FA: Folic acid
