# Supplementary material for: Autophagy impairment in patients with obstructive sleep apnea modulates intermittent hypoxia-induced oxidative stress and cell apoptosis via hypermethylation of the ATG5 gene promoter region
Source: Eur J Med Res. 2023 Feb 17;28:82. doi: 10.1186/s40001-023-01051-4 (PMC9936724; doi:10.1186/s40001-023-01051-4)
Supplement: Supplementary file 1 — Additional file 1: Table S1. Primer sequences for assaying quantitative real-time polymerase chain reactions and pyro-sequencing used in the present study. Figure S1. Pyrograms of the representative CpG sites assayed of the LC3B gene promoter region. Figure S2. Pyrograms of the representative CpG sites assayed of the ATG5 gene. Figure S3. Pyrograms of the representative CpG sites assayed of the p62 gene. Figure S3. Pyrograms of the representative CpG sites assayed of the p62 gene. Figure S4. Effects of rapamycin (Rap) and mesenchymal stem cell (MSC) treatment on late apoptosis and autophagy related gene (ATG) expressions under intermittent hypoxia with re-oxygenation stimuli (IHR) in THP-1 cells. [file 40001_2023_1051_MOESM1_ESM.docx]

**Additional file**

Autophagy impairment in patients with obstructive sleep apnea modulates intermittent hypoxia-induced oxidative stress and cell apoptosis via hypermethylation of the ATG5 gene promoter region

Yung-Che Chen^1, 2, 3*^, I-Chun Lin^4^, Mao-Chang Su^1, 3, 5^, Po-Yuan Hsu^1^, Chang-Chun Hsiao^1, 6^, Te-Yao Hsu^7^, Chia-Wei Liou^8^, Yu-Mu Chen^1, 3^, Chien-Hung Chin^1, 3^, Ting-Ya Wang^1^, Jen-Chieh Chang^7^, Yong-Yong Lin^1^, Chiu Ping Lee^1^, Meng-Chih Lin^1, 2, 3*^

**Table S1. Primer sequences for assaying quantitative real-time polymerase chain reactions and pyro-sequencing used in the present study.**

| **Gene name** | **Primer sequences** | **5’ to 3’** |
| --- | --- | --- |
| *BECN 1* | Forward | GGTGTCTCTCGCAGATTCATC |
|  | Reverse | TCAGTCTTCGGCTGAGGTTCT |
| *ATG5* | Forward | AAAGATGTGCTTCGAGATGTG |
|  | Reverse | CACTTTGTCAGTTACCAACGTCA |
| *LC3B* | Forward | GAGAAGCAGCTTCCTGTTCTGG |
|  | Reverse | GTGTCCGTTCACCAACAGGAAG |
| *p62* | Forward | GCACCCCAATGTGATCTGC |
|  | Reverse | CGCTACACAAGTCGTAGTCTGG |
| *ULK1* | Forward | GGCAAGTTCGAGTTCTCCCG |
|  | Reverse | CGACCTCCAAATCGTGCTTCT |
| *ATG9A* | Forward | CTGCCCTTCCGTATTGCAC |
|  | Reverse | CTCACGTTTGTGGATGCAGAT |
| *GAPDH* | Forward | 5'-GAAGAGCCAAGGACAGGTAC |
|  | Reverse | 5'-CAACTTCATCCACGTTCACC |
| *ATG5* pyrosequencing |  |  |
| Region 1 (-172 to -159 from TSS) | Forward PCR | GATGTTAGGAAGAAAGGGATGTT |
|  | Reverse PCR | Biotin-AACTAAACCCTACTACACTTCC |
|  | Forward sequencing | AAGTTAATTTTTTTAAGTTTTAATA |
| Region 2 (-108 to -61 from TSS) | Forward PCR | GATGTTAGGAAGAAAGGGATGTT |
|  | Reverse PCR | Bioton-AACTAAACCCTACTACACTTCC |
|  | Forward sequencing | AGGGTAGAAGTTATGAAT |
| Region 3 (-32 to +31 from TSS) | Forward PCR | GATGTTAGGAAGAAAGGGATGTT |
|  | Reverse PCR | Biotin-AACTAAACCCTACTACACTTCC |
|  | Forward sequencing | GTTGGGTTAGGTAGAAT |
| Region 4 (+44 to +96 from TSS) | Forward PCR | GATGTTAGGAAGAAAGGGATGTT |
|  | Reverse PCR | Biotin-AACTAAACCCTACTACACTTCC |
|  | Forward sequencing | GGGTGATTGGATTTG |
| *LC3B* pyrosequencing, region 1 (-172 to -118 bp from TSS) | Forward PCR | GTATTATTAAGTTTTTTTGGAGGGGAAAG |
|  | Reverse PCR | Biotin-CACTTCCCTTATATCTCCTCAAC |
|  | Forward sequencing | GTTGTTATTTTTTTAGGGT |
| *LC3B* pyrosequencing, region 2 (-97 to -22 bp from TSS) | Forward PCR | GTATTATTAAGTTTTTTTGGAGGGGAAAG |
|  | Reverse PCR | Biotin-CACTTCCCTTATATCTCCTCAAC |
|  | Forward sequencing | GTTATTTTAGGGGTTTTA |
| *p62* pyrossequencing | Forward PCR | Biotin-GGGGTAGTAGGTAGTAGGGT |
| (-164 to -74 bp from TSS) | Reverse PCR | ACCCTCCCCCACTTATTT |
|  | Forward sequencing | CCCCACTTATTTACTACTAA |


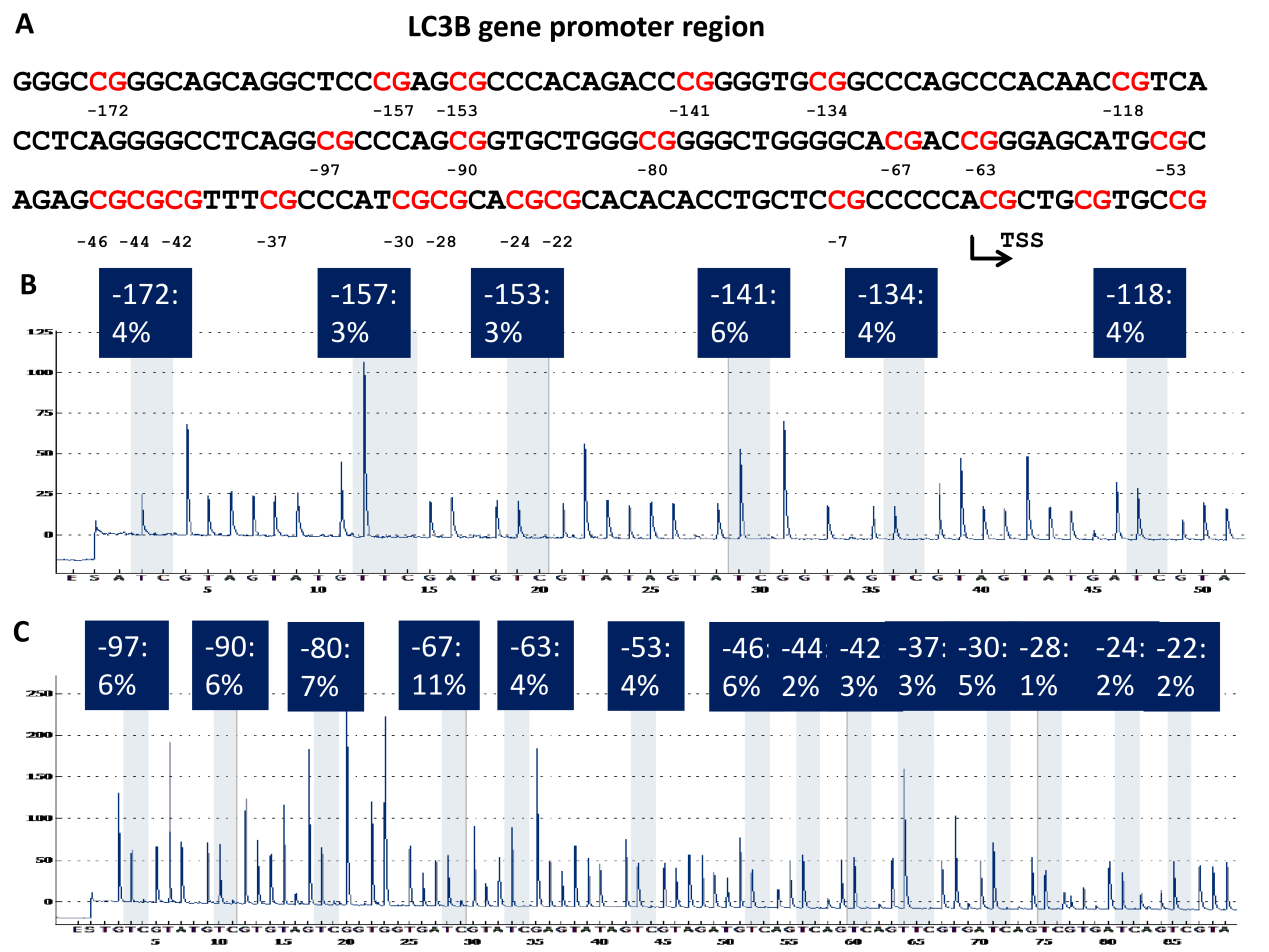


**Figure S1. Pyrograms of the representative CpG sites assayed of the LC3B gene promoter region.** (A) DNA sequence of the *LC3B* gene promoter region. Representative pyrograms show the percentage of DNA methylation levels over (B) -172, -157, -153, -141, -134, and -118 CpG sites of the *LC3B* gene, and (C) over -97, -90, -80, -67, -63, -53, -46, -44, -42, -37, -30, -28, -24, and -22 CpG sites of the *LC3B* gene in the peripheral blood mononuclear cell sample from an OSA patient.

**
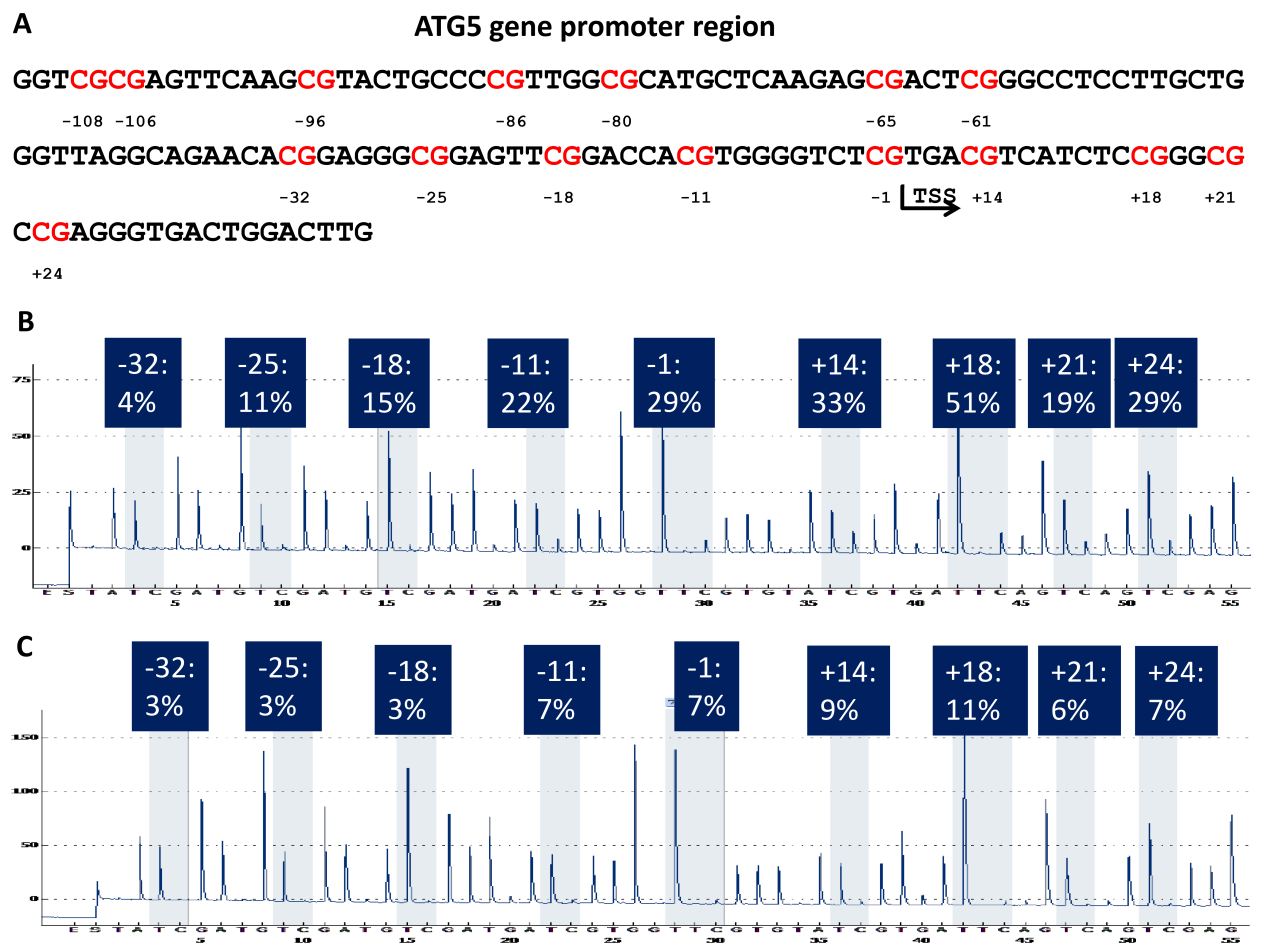
**

**Figure S2. Pyrograms of the representative CpG sites assayed of the *ATG5* gene.** (A) DNA sequence of the *ATG5* gene promoter region. Representative pyrograms show the percentage of DNA methylation levels over -32, -25, -18, -11, -1, +14, +18, +21, and +24 CpG sites of the *ATG5* gene (B) in the peripheral blood mononuclear cell sample from an OSA patient, and (C) in THP-1 cells under intermittent hypoxia with re-oxygenation stimuli.


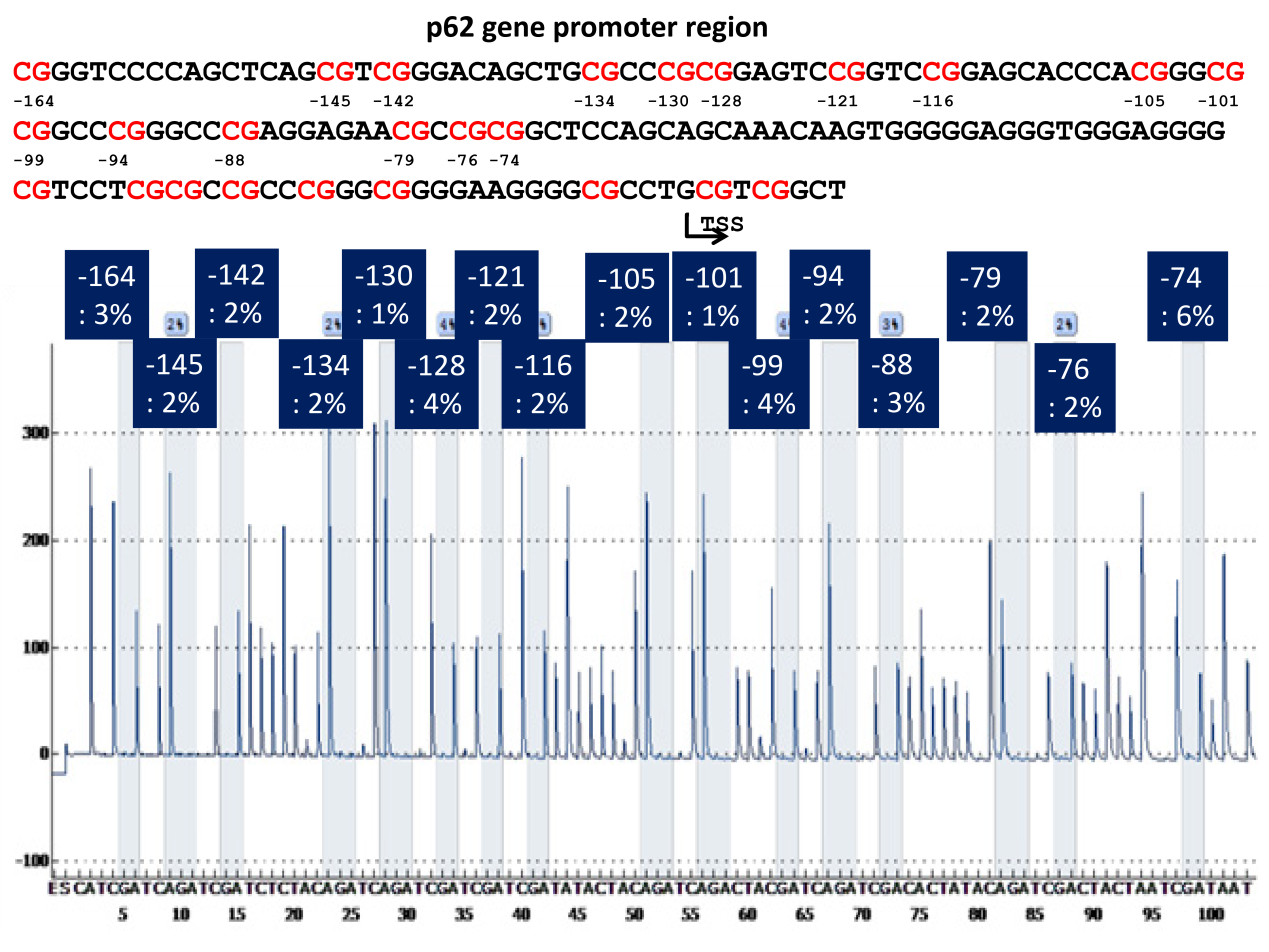


**Figure S3. Pyrograms of the representative CpG sites assayed of the *p62* gene.** (A) DAN sequence of the *p62* gene promoter region. (B) Representative pyrograms show the percentage of DNA methylation levels over -164, -145, -142, -134, -105, -101, -99, -94 CpG sites of the *p62* gene in the peripheral blood mononuclear cell sample from an OSA patient.


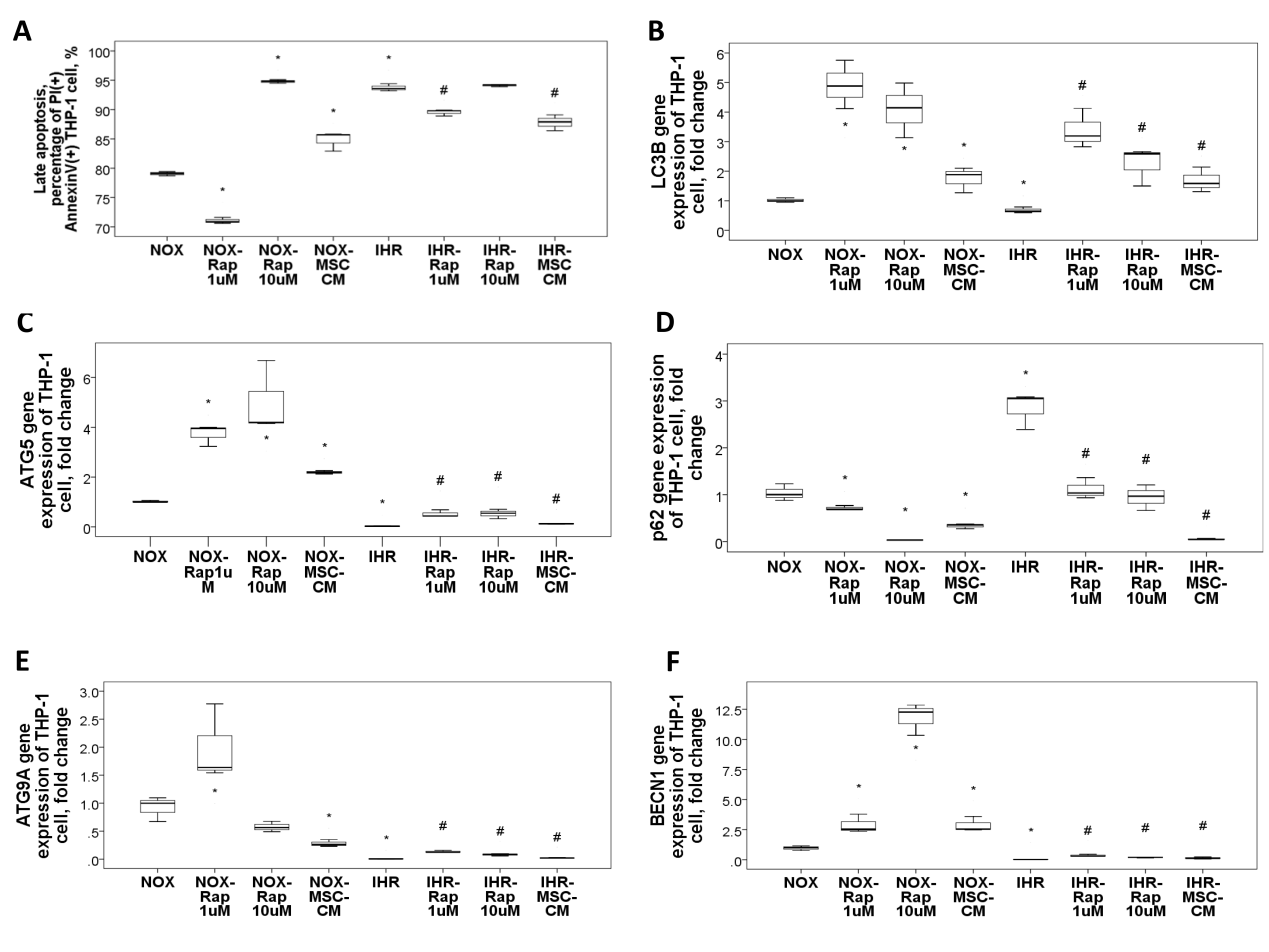


**Figure S4. The effects of rapamycin (Rap) and mesenchymal stem cell (MSC) treatment on late apoptosis and autophagy related gene (ATG) expressions under intermittent hypoxia with re-oxygenation stimuli (IHR) in THP-1 cells.** (A) Late apoptosis was increased with IHR stimuli, and partly reversed with 1 μM Rap or MSC-condition medium (CM) treatment. Either Rap or MSC-CM treatment reversed IHR-induced down-regulations of the (B) *LC3B* and (C) *ATG5* genes, and (D) *p62* up-regulation. Both treatments also partly reversed IHR-induced down-regulations of the (E) *ATG9A* and (F) *BECN1* genes.
